# LESSONS LEARNED FROM SAVVY EXPRESS: A FEASIBILITY STUDY OF A PSYCHODUCATIONAL INTERVENTION FOR CARE PARTNERS

**DOI:** 10.1093/geroni/igz038.1054

**Published:** 2019-11-08

**Authors:** Maria P Aranda, Debra Cherry

**Affiliations:** 1 University of Southern California, Los Angeles, California, United States; 2 Alzheimer’s Los Angeles, Los Angeles, California, United States

## Abstract

An increasing number of families, funders, and community providers seek very brief psychosocial caregiver interventions, yet the evidence for such condensed interventions is not established. Based on the Savvy Caregiver Program, we explored the feasibility, acceptability, and outcome trends for a condensed 3-session version titled, Savvy Express. Based on a single-group, pre- and post-test intervention design, we examined post-intervention and 3-month data on 116 English-speaking racially and ethnically diverse care partners caring family members with Alzheimer’s disease and related dementias. 41% of the sample was non-Latino white and comprised of Latinas, African Americans and Asian American/API. Most care partners were either adult children or spouses caring for someone with AD or other dementia. Over 80% were college educated. Two of three participants completed all 3 classes. Our findings indicate significant improvements in caregiver levels of depressive symptomatology and anxiety, competence, management of the situation, reduction of expectations, making positive comparisons, and reactivity to the family member’s memory behavior. Upwards of 90% would recommend the program to other caregivers. Savvy Express is a brief caregiver intervention with high acceptability and feasibility. Improvements in care partner psychosocial outcomes signal a promising practice to reduce the burden of caregiving. A major focus of the paper focuses on barriers and facilitators to uptake of the study procedures and intervention with community-based partners. Future work is needed to establish the efficacy of Savvy Express across a longer observation period, and with less educated, low-income participants, and limited English-speaking families.

